# Effects of Human Parvovirus B19 and Bocavirus VP1 Unique Region on Tight Junction of Human Airway Epithelial A549 Cells

**DOI:** 10.1371/journal.pone.0107970

**Published:** 2014-09-30

**Authors:** Chun-Ching Chiu, Ya-Fang Shi, Jiann-Jou Yang, Yuan-Chao Hsiao, Bor-Show Tzang, Tsai-Ching Hsu

**Affiliations:** 1 Institute of Microbiology and Immunology, Chung Shan Medical University, Taichung, Taiwan; 2 Department of Neurology and Department of Medical Intensive Care Unit, Chunghua Christian Hospital, Chunghua, Taiwan; 3 Department of Biomedical Sciences, Chung Shan Medical University, Taichung, Taiwan; 4 Department of Biochemistry, School of Medicine, Chung Shan Medical University, Taichung, Taiwan; 5 Institute of Biochemistry and Biotechnology, Chung Shan Medical University, Taichung, Taiwan; 6 Clinical Laboratory, Chung Shan Medical University Hospital, Taichung, Taiwan; Faculty of Biochemistry Biophysics and Biotechnology, Jagiellonian University, Poland

## Abstract

As is widely recognized, human parvovirus B19 (B19) and human bocavirus (HBoV) are important human pathogens. Obviously, both VP1 unique region (VP1u) of B19 and HBoV exhibit the secreted phospholipase A2 (sPLA2)-like enzymatic activity and are recognized to participate in the pathogenesis of lower respiratory tract illnesses. However, exactly how, both VP1u from B19 and HBoV affect tight junction has seldom been addressed. Therefore, this study investigates how B19-VP1u and HBoV-VP1u may affect the tight junction of the airway epithelial A549 cells by examining phospholipase A2 activity and transepithelial electrical resistance (TEER) as well as performing immunoblotting analyses. Experimental results indicate that TEER is more significantly decreased in A549 cells by treatment with TNF-α (10 ng), two dosages of B19-VP1u and BoV-VP1u (400 ng and 4000 ng) or bee venom PLA2 (10 ng) than that of the control. Accordingly, more significantly increased claudin-1 and decreased occludin are detected in A549 cells by treatment with TNF-α or both dosages of HBoV-VP1u than that of the control. Additionally, more significantly decreased Na+/K+ ATPase is observed in A549 cells by treatment with TNF-α, high dosage of B19-VP1u or both dosages of BoV-VP1u than that of the control. Above findings suggest that HBoV-VP1u rather than B19 VP1u likely plays more important roles in the disruption of tight junction in the airway tract. Meanwhile, this discrepancy appears not to be associated with the secreted phospholipase A2 (sPLA2)-like enzymatic activity.

## Introduction

Human parvovirus B19 (B19) is a significant human pathogen that belongs to the *Parvoviridae* family [1]. B19 DNA or antigen has been found in various human tissues, implying the possible existence of comprehensive B19-infectious targets [2]. As the pathogen of the fifth disease, B19 is more frequently associated with hematological symptoms and arthropathy, leading to severe diseases during pregnancy [3]–[5]. Also implicated as a trigger of various autoimmune diseases [6]–[7], the B19 virus also occasionally occurs in the respiratory tract [3]–[5]. The icosahedral capsid of B19 consists of two structural proteins (i.e. VP1 (83 kDa) and VP2 (58 kDa)), which are identical except for 227 amino acids at the amino-terminal end of the VP1-protein, commonly referred to as the VP1-unique region (VP1u) [1]. In recent decades, the phospholipase A2 (PLA2)-like activity of B19-VP1u has been identified [8] and associated with its infectivity and pathogenesis of various diseases [9]–[12].

As a newly discovered human parvovirus identified by Allander *et al.* in 2005, human bocavirus (HBoV) belongs to the *Parvovirida* family as B19 and is most likely the second known parvovirus species pathogenic to humans [13]. HBoV contains a 5.3-kb single-stranded DNA and the genome polarity is negative [14], which encodes two non-structural proteins NS1 and NP1, and two structural proteins VP1 and VP2. The VP1 of HBoV has an amino-acid sequence identical to that of the VP2 protein, except for additional 129 amino acids at its amino terminus, commonly referred to as the VP1 unique region (VP1u) [15]–[16]. Similar to B19 virus, HBoV-VP1u also has a PLA2 motif and demonstrated to have sPLA2 activity [17]. HBoV has been linked to upper and lower respiratory tract diseases and gastroenteritis worldwide. The HBoV infection has various clinical symptoms, including coughing, pharyngitis, wheezing, dyspnea, rhinitis, acute otitis media, fever, pneumonia, diarrhea, vomiting and nausea [14]. According to a recent study, HBoV infects polarized primary human airway epithelia, leading to the characteristic airway epithelial damage [18]–[20]. However, the precise mechanism and role of PLA2 activity of HBoV in airway epithelial damage remain unclear.

As is widely recognized, the epithelium in the respiratory system and other organs functions as a selective gate between the external environment and underlying tissue. These epithelial cells are polarized by the formation of specialized cell-cell junctions, which are referred to as the apical junction complex such as adherent junctions (AJs) and tight junctions (TJs) [21]. TJs are close cell–cell connections that form paired strands, which seal the space between neighboring cells and control the interactive permeability of small molecules [22]. TJs also function as a barrier to potential pathogens and foreign particles, preventing infection and tissue injury [23]. TJs consist mainly of a multi-protein complex containing the tetraspanin claudins, occludin and cytosolic proteins such as zona occludens (ZO), which links the cytoskeletal assembly to the TJ membrane [22]. Owing to its role in defending the infection, epithelia in the respiratory tract is vulnerable to molecules with proteolytic activity such as sPLA2 [24]. Although sPLA2 of B19 and HBoV have been implicated in a wide range of cellular responses [25]–[26], exactly how human parvovirus and their sPLA2 affect the tight junction in airway epithelial cells is relatively unknown. By using A549 cells [27], a well-known in vitro model of TJs, this study investigates how B19-VP1u and HBoV-VP1u affect tight junction molecules.

## Materials and Methods

### Preparation of recombinant human HBoV- VP1u and B19-VP1u proteins

A 387-bp DNA fragment encompassing nucleotides 3056–3442 of the Taiwan HBoV strain [28] (TW125_07: GeneBank accession number EU984241.1, provided by Centers for Disease Control, Taipei, Taiwan) was amplified by the polymerase chain reaction using primers 5′-GCGAATTCATGCCTCCAATTAAG-3′ (forward) and 5′-GCGTCGACTGAGGTTCCTG G-3′ (reverse), which were introduced a *EcoRI* site at the 5′ end and a *Sal I* site at the 3′ end for cloning into pET-32a. The amplification was performed in a 50 µl reaction volume containing 10× reaction buffer (Promega, Madison, WI), 1.5 µmol/l of MgCl2, 200 µmol/l of dNTPs, 1 µmol/l of each primer and 2.5 units of Taq DNA polymerase (Promega) using a GeneAmp PCR system2400 (Perkin-Elmer, Foster City, CA). After an initial denaturation step of 5 min at 94°C, 30 cycles were performed at 94°C for 45 s, 56°C for 45 s, and 72°C for 1 min. The amplification PCR products were subjected to electrophoresis on a 1% agarose gel. The ligatant, so called pET32a-HBoV-VP1u was then transformed into Escherichia coli BL21-DE3 competent cells, which were obtained from Invitrogen (Carlsbad, CA). Restriction enzyme digestion and DNA sequencing analysis were used to verify the plasmid. E. coli (BL21-DE3) clones containing B19-VP1u [25] or HBoV-VP1u cDNA in pET-32a expression vector (Novagene, Cambridge, MA) were grown overnight in one liter L-Broth containing 100 ug/ml ampicillin at 37°C with shaking. When the OD 600 reached 0.7–0.9, protein expression was induced by addition of IPTG to a concentration of 1 mM and incubated for another 3 hr. The cells were harvested by centrifugation at 4000 g for 20 min and resuspended in 20 ml sonication buffer (50 mM NaPO4 pH 8/0.25 mM EDTA). Lysozyme was added to a final concentration of 1 mg/ml and kept on ice for 30 min. The cells were sonicated (W385, Heat systems-ultrasonic, INC) for a total of 30 min at 5 min intervals, centrifuged 10,000 g for 30 min. The pellet was dissolved with 10 ml buffer B (8 M urea; 0.1 M NaH2PO4; 0.02 M Tris-HCl; pH 8.0) for 1 hr at room temperature, and centrifuge lysate at 10,000 g for 30 min at room temperature to pellet the cellular debris. The supernatant was loaded onto a Ni-NTA spin column (Qiagen, Chatsworth, CA, USA) or Nickel Magnetic Beads (Millpore, MA, USA), and purified specific proteins.

### sPLA2 catalytic activity

B19-VP1u and HBoV-VP1u proteins were assayed for sPLA2 activity by using a colorimetric assay (sPLA2 Activity Kit; Cayman Chemical), in accordance with the manufacturer’s instructions, with dynamic colorimetric measurements (the optical density at 414 nm) determined every minute for 10 min. Results are revealed as micromoles per minute per milliliter.

### Cell culture

Human airway epithelial A549 cells were originally obtained from American type culture collection (ATCC) and were grown in Dulbecco’s modified Eagle medium (DMEM) supplemented with 10% fetal bovine serum (FBS) (GIBCO-BRL) at 37°C and 5% CO_2_ incubator. For experiments, A549 cells were seeded in 100-cm^2^ dishes and grown to 100% confluence in prior to different treatments. The effect of B19-VP1u and HBoV-VP1u at different dosages on tight junction of A549 cells was performed.

### Measurement of Transepithelial Electrical Resistance (TEER)

To assess epithelial barrier integrity, A549 cells were plated and cultured for 2 days and then exposed to TNF-α (10 ng/ml) [29], bee venom PLA2 (1 ug/ml), 400 ng/ml and 4000 ng/ml recombinant B19-VP1u or HBoV-VP1u proteins for 24 h, respectively. TEER (Ω.cm^2^) was measured with an epithelial voltmeter (Millicell ERS-2 (Millipore-ERS, Millipore, MA, USA), Millipore, MA, USA) using chopstick-like electrodes as a measurement of tight junctional barrier formation [19]–[20]. For experiments, cells were seeded at high density on Snapwell inserts (Costar, Corning, MA, USA) and maintained at 37°C in a 5% CO_2_, 95% air atmosphere. TEER values were obtained by subtracting blank filter resistance from all readings.

### Cell lysis and Protein Extraction

Cultured cells were collected by centrifugation (800 g×5 min) and washed by ice-cold PBS twice. The collected cells were resuspended in 600 ul of PRO-PREP™ buffer (iNtRON Biotech, Gyeonggi-do, Korea) and stood on ice for 60 min. The samples were then centrifuged at 13,000 rpm for 5 min at 4°C, the supernatant regarded as crude extract was transferred into a new eppendorf and stored at –20°C. Protein concentration of the samples was determined by a modified Bradford’s assay using a spectrophotometer (Hitachi U3000, Tokyo, Japan) at 595 nm and BSA as standard.

### Immunoblotting

Protein samples were separated in 12.5 or 10% of SDS-PAGE and electrophoretically transferred to nitrocellulose membrane (Amersham Biosciences, Piscataway, NJ, USA). After blocking with 5% non-fat dry milk in PBS, antibodies against claudin-1, occludin (Invirtrogen, CA, USA), Na/K+ ATPase (Santa Cruz Biotechnology, CA, USA) and actin (Upstates, Charlottesville, Virginia, USA) were diluted in PBS with 2.5% BSA and incubated for 1.5 hr with gentle agitation at room temperature. The membranes were then incubated with horseradish peroxidase (HRP) conjugated secondary antibody. Immobilon Western Chemiluminescent HRP Substrate (Millipore, MA, USA) and chemiluminescence imaging analyzer (GE ImageQuant TL 8.1, GE Healthcare Life Sciences, PA, USA) were used to detect the antigen-antibody complexes. The blots were then quantified by densitometric apparatus (Appraise, Beckman-Coulter, Brea, California, USA).

### Statistical analysis

All of the statistical analyses were performed using GraphPad Prism 5 software (GraphPad Software, CA) by one-way analysis of variance (One-way ANOVA) followed by Tukey multiple-comparisons test. Data were represented as mean ± SEM and verified at least three independent experiments. A value of P<0.05 was considered statistically significant. The significant differences were stressed with symbols as shown in figures.

## Results

### Recombinant B19-VP1u and HBoV-VP1u proteins reveal sPLA2 activity

As is well known, B19-VP1u and HBoV-VP1u play important roles in infectivity and pathogenesis for various diseases, possibly owing to their PLA2 enzymatic activities. This study attempted to confirm whether B19-VP1u and HBoV-VP1u proteins have the PLA2 activity by constructing and purifying the recombinant B19-VP1u and HBoV-VP1u proteins, as described in the Materials and Methods Section, in order to analyze the sPLA2 activity. Table 1 summarizes the results of sPLA2 activities in TNF-α, bee venom PLA2, B19-VP1u and HBoV-VP1u. As a positive control, bvPLA2 revealed PLA2 enzymatic activity with a value of 0.235±0.003 µmol/min/mL, whereas no PLA2 activity was detected in TNF-α. Notably, significant PLA2 activity was detected in both recombinant B19-VP1u and HBoV-VP1u proteins with a PLA2 activity of 0.035±0.002 µmol/min/mL and 0.065±0.005 µmol/min/mL, respectively. Accordingly, more significantly increased sPLA2 activity was observed in high dosage (4000 ng) of recombinant B19-VP1u and HBoV-VP1u proteins with an activity of 0.765±0.012 µmol/min/mL and 1.362±0.019 µmol/min/mL, respectively.

**Table 1:** Secreted phospholipase A2 (sPLA2) activity of recombinant B19-VP1u or HBoV-VP1u proteins.

Proteins	sPLA2 activity (µmol/min/mL)
TNF-α (10 ng)	ND
bvPLA2 (10 ng)	0.235±0.003
B19-VP1u (400 ng)	0.035±0.002
B19-VP1u (4000 ng)	0.765±0.013
HBoV-VP1u (400 ng)	0.065±0.005
HBoV-VP1u (4000 ng)	1.362±0.019

bvPLA2: sPLA2 from bee venom PLA2 control; ND: no detected.

### Effects of Recombinant B19-VP1u and HBoV-VP1u proteins on A549 epithelial tight junction permeability

**Figure 1:**
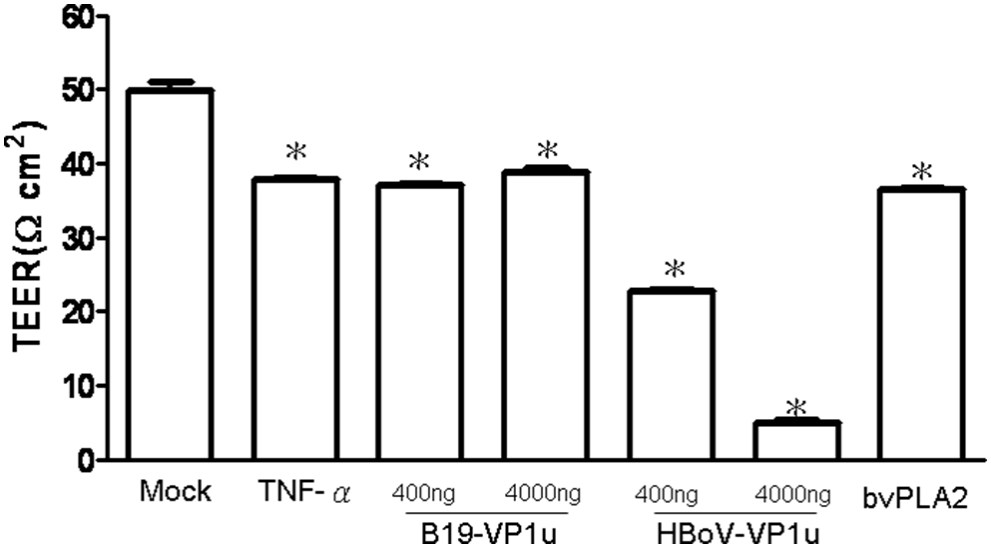
Effects of human parvovirus proteins on transepithelial electrical resistance (TER). A549 cells treated with PBS (mock), TNF-α (10 ng/ml), two dosages of recombinant B19-VP1u and HBoV-VP1u (400 ng/ml and 4000 ng/ml) and bee venom PLA2 (10 ng/ml) were used to detected the electrical resistance. Similar results were observed in three independent experiments and * indicates the significant difference as compared to the mock, *P*<0.05.

Since a decline in TEER across the monolayer of cells reflecting increases permeability, this study conducted TEER experiments on TJs of A549 cells by treating with TNF-α, bee venom PLA2, B19-VP1u and HBo-VP1u, respectively. Significant declines in TEER were detected in A549 cells monolayer during the treatment of TNF-α, bee venom PLA2, B19-VP1u and HBo-VP1u as compared to the mock control [Fig. 1]. Interestingly, only treatment with HBo-VP1u revealed a dose-dependent and a more significant decline in TEER. This finding indicates an increased permeability on TJs in A549 cells.

### Effect of Recombinant B19-VP1u and HBoV-VP1u proteins on A549 epithelial tight junction molecules

**Figure 2:**
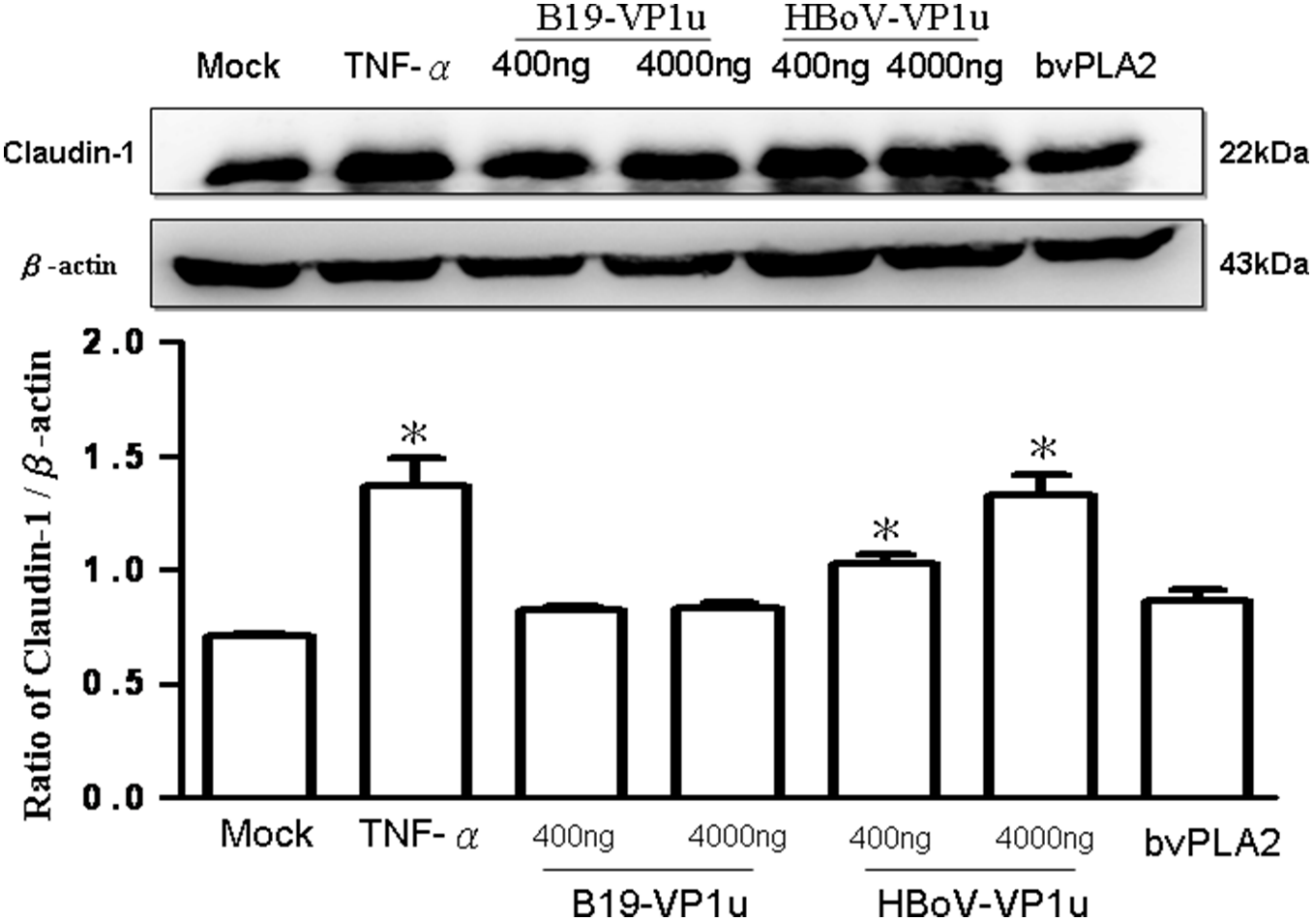
Expression of claudin-1. Cell lysates obtained from A549 cells treated with PBS (mock), TNF-α (10 ng/ml), two dosages of recombinant B19-VP1u and HBoV-VP1u (400 ng/ml and 4000 ng/ml) and bee venom PLA2 (10 ng/ml) were probed with antibodies against claudin-1. Quantified result was shown in the lower panel. Similar results were observed in three independent experiments and * indicates the significant difference as compared to the mock, *P*<0.05.

**Figure 3:**
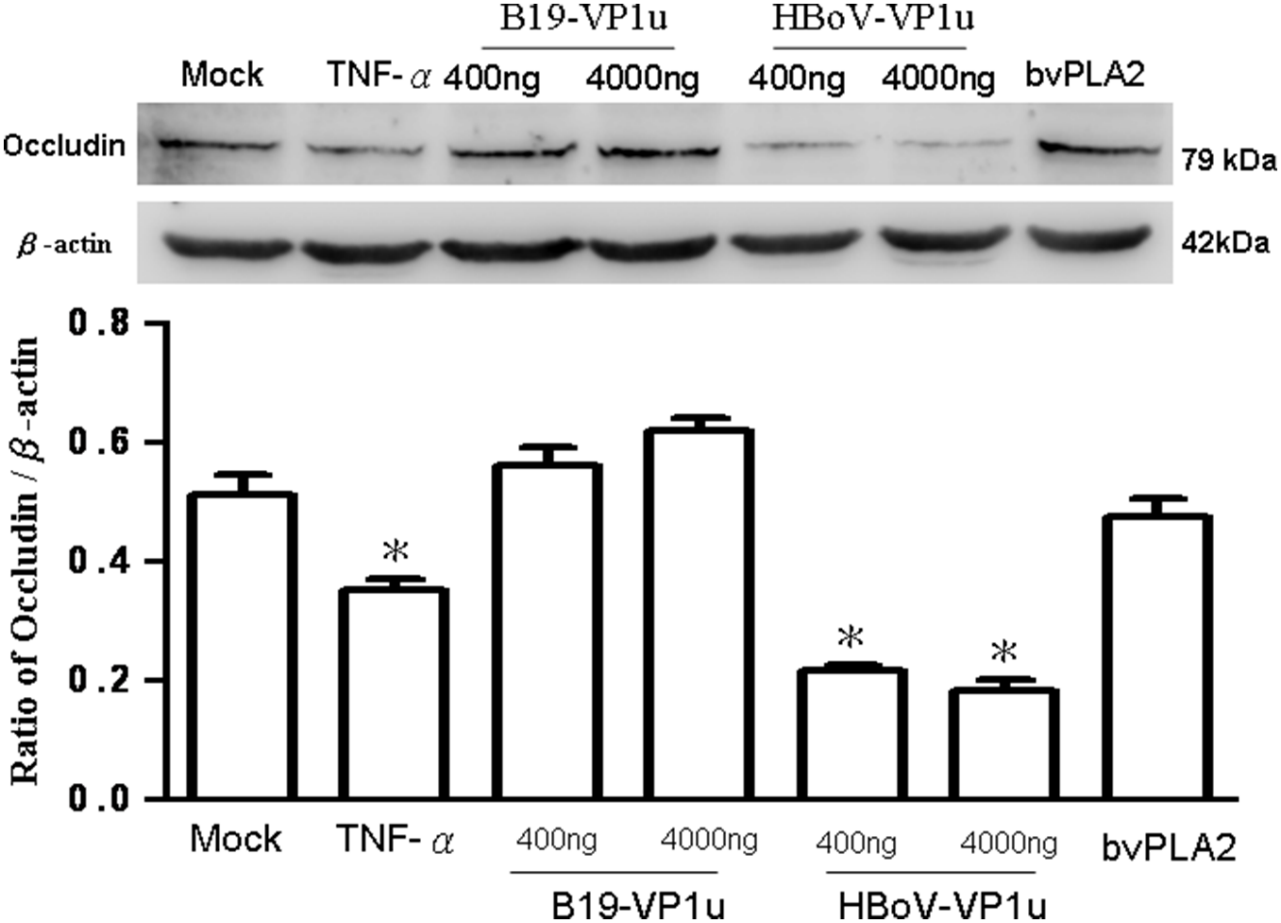
Expression of occludin. Cell lysates obtained from A549 cells treated with PBS (mock), TNF-α (10 ng/ml), two dosages of recombinant B19-VP1u and HBoV-VP1u (400 ng/ml and 4000 ng/ml) and bee venom PLA2 (10 ng/ml) were probed with antibodies against occludin. Quantified result was shown in the lower panel. Similar results were observed in three independent experiments and * indicates the significant difference as compared to the mock, *P*<0.05.

The effects of both B19-VP1u and HBoV-VP1u on TJs in A549 cells were more thoroughly investigated by examining two important indicators of TJs (i.e. claudin-1 and occludin) by using immunoblotting analysis. According to those results, claudin-1 significantly increased in A549 cells by treating with TNF-α and both dosages of HBoV-VP1u as compared to the controls (Fig. 2). Conversely, claudin-1 did not significantly vary in A549 cells by treating with bee venom PLA2 and both dosages of B19-VP1u (Fig. 2). Accordingly, similar results were observed in the expression of occludin. The protein levels of occludin in A549 cells were more significantly decreased in response to TNF-α or HBoV-VP1u treatments than in the controls, whereas no significant variation was detected in A549 cells when treating with B19-VP1u or bee venom PLA2 (Fig. 3).

### Effect of Recombinant B19-VP1u and HBoV-VP1u proteins on A549 epithelial Na+/K+ ATPase

**Figure 4:**
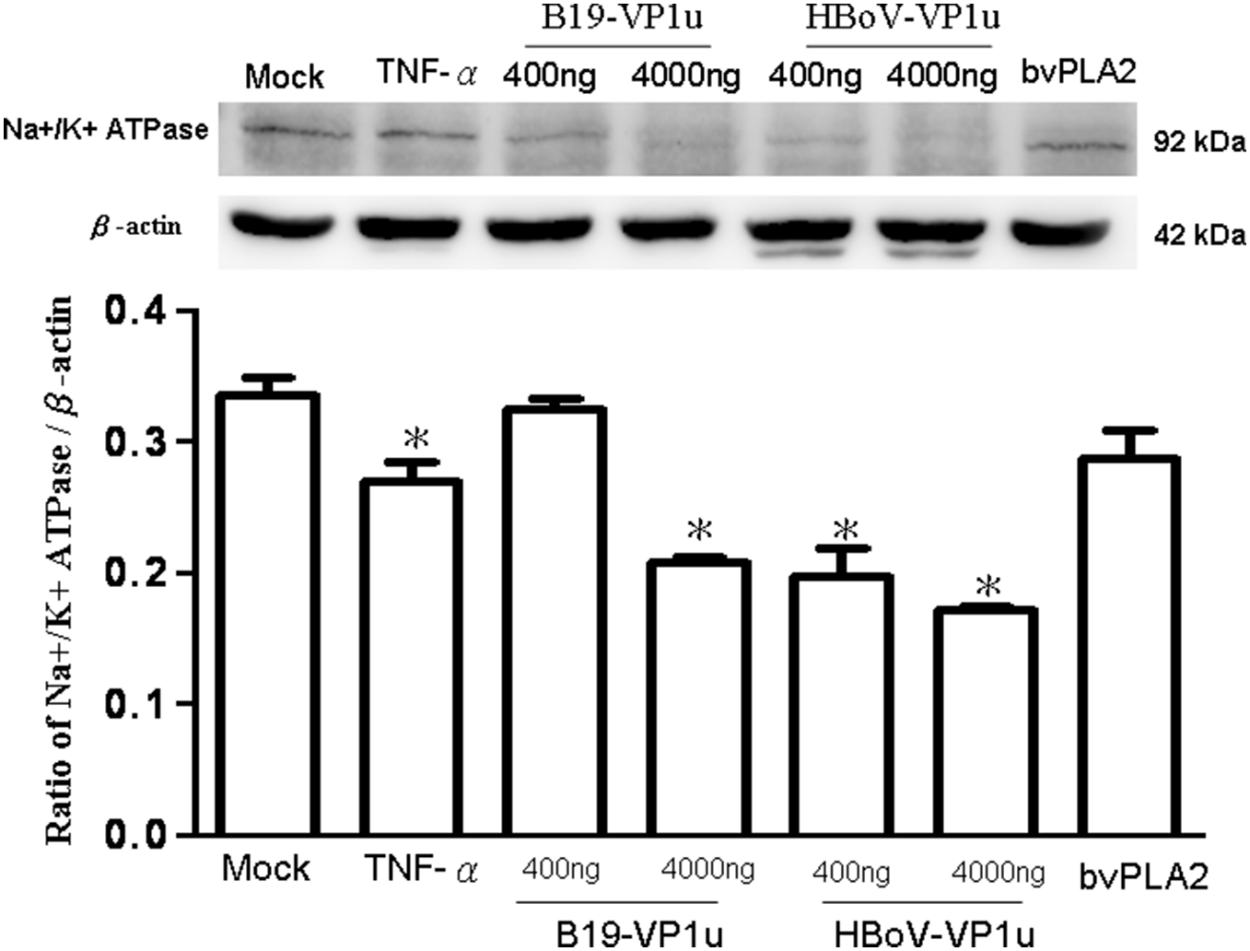
Expression of Na+/K+ ATPase. Cell lysates obtained from A549 cells treated with PBS (mock), TNF-α (10 ng/ml), two dosages of recombinant B19-VP1u and HBoV-VP1u (400 ng/ml and 4000 ng/ml) and bee venom PLA2 (10 ng/ml) were probed with antibodies against Na+/K+ ATPase. Quantified result was shown in the lower panel. Similar results were observed in three independent experiments and * indicates the significant difference as compared to the mock, *P*<0.05.

As is well known, the expression of Na+/K+ ATPase is closely related to cell tight junction and polarity of epithelial cells. Therefore, this study examined the expression of the Na+/K+ ATPase in A549 cells when treating with recombinant B19-VP1u and HBoV-VP1u proteins by immunoblotting analysis. More significant decreases of Na+/K+ ATPase in A549 cells were detected by treatment with TNF-α, 4000 ug/ml B19-VP1u and both dosages of HBoV-VP1u than that of the controls (Fig. 4). Conversely, Na+/K+ ATPase in A549 cells did not significantly vary by treatment with 400 ug/ml B19-VP1u and bee venom PLA2 as compared to the controls (Fig. 4).

## Discussion

As a significant human pathogen, B19 is associated with the pathogenesis of many diseases, including respiratory disorders [1], [30]. B19 DNA or antigen has been found in various human tissues, including the respiratory tract [2]. Meanwhile, parvovirus B19 was also detected in the lower respiratory tract [5], which appears to be the most common route of transmission for B19 [3]–[4]. Additionally, as a newly discovered human parvovirus in respiratory tract samples, human bocavirus (HBoV) is also associated with the pathogenesis of respiratory tract diseases [15]. According to a recent study of pediatric lower respiratory tract infection, the average incidence of HBoV infection in a subtropical area of China is 6.6% [31]. Similarly, another study involving the detection of HBoV in children with upper respiratory tract infection found an incidence of 4.8% [32]. Although both B19 and HBoV are strongly associated with the infection in respiratory tract, little is known about the possible mechanism on disrupting the TJs. This study first described the disruptive effects of HBoV-VP1u rather than B19-VP1u on TJs of A549 cells by decreasing the expression of occludin and Na+/K+ ATPase as well as the significantly decreased TEER and increased claudin-1 expression. Above findings suggest that HBoV-VP1u rather than B19 VP1u is likely to play more important roles in the disruption of a tight junction in an airway tract.

Secretory phospholipases A2 (sPLA2) consist of a large and widely distributed family of enzymes that are distributed throughout the epidermis [33]–[34]. These enzymes hydrolyze the glycerophospholipid ester bond at the sn-2 position to generate a free fatty acid and a lysophospholipid [34]. The actions of sPLA2 are involved in several essential epidermal processes, and the most extensively investigated action is the role of sPLA2s in inflammation [35]. Correspondingly, several types of secretory phospholipase A2 (sPLA2) are expressed in lung tissue, yielding various eicosanoids that cause pulmonary edema [36]. As is well known, the secretion of enzymes and cytokines induced by sPLA2s from human macrophages plays important roles in inflammation and tissue damage associated with the release of sPLA2s [37]. Induction of sPLA2 has also been observed in animal models of acute lung injury [38]–[39]. According to a related study, sPLA2 plays a unique role in checkpoint control of acute inflammation as well as an epithelial regulator of inflammation in asthma patients [35], [40]. Both (VP1 unique region (VP1u) of B19 and HBoV exhibit the secreted phospholipase A2 (sPLA2)-like enzymatic activity and are suggested to participate in the pathogenesis of lower respiratory tract illnesses [3]–[5], [20]. However, the pathogenic effect of their PLA2-like enzymatic activity on epithelia barrier of respiratory tract is still unclear. As is well known, TNF-α has disruptive effects on TJs, which could decrease the value of TEER, expression of occludin and Na+/K+ ATPase, as well as increase the expression of claudin-1 [41]. Therefore, in this study, TNF-α was used as a positive control on TJs disruption. According to our results, bvPLA2, both dosages of B19-VP1u and HBoV-VP1u all exhibited the enzymatic activity of sPLA2 and significantly reduced transepithelial electrical resistance, implying an increased permeability of TJs in A549 cells. Although high dosage (4000 ng/ml) of B19-VP1u could significantly reduced the expression of Na+/K+ ATPase, only TNF-α and HBoV-VP1u significantly increased the expression of accludin-1 and significantly decreased the expressions of occludin and Na+/K+ ATPase. These findings strongly suggest that HBoV-VP1u rather than B19-VP1u more significantly impacted TJs disruption in A549 cells, and is not likely associated with their sPLA2-like enzymatic activities.

Although both B19 and HBoV have been closely linked to the infection and injury in air-tract illnesses, the pathogenic mechanism of these viruses is still relatively unknown. This study first demonstrated that HBoV-VP1u revealed a significantly disruptive effect on TJs in A549 cells whereas B19-VP1u exhibited a markedly lower ability in terms of TJs disruption. Additionally, the disruptive effect of HBoV-VP1u is likely not associated with its sPLA2-like enzymatic activity. Based on the above findings, we believe that B19-VP1u and HBoV-VP1u might function in different roles in respiratory infection. Efforts are underway to more thoroughly elucidate the precise mechanism.
